# Relationship between serum vitamin D and thyroid hormone profiles in male patients with alcohol dependence

**DOI:** 10.1186/s12888-023-05274-x

**Published:** 2023-10-24

**Authors:** Yu Liu, Yayun Xu, Yongmei Wang, Liangjun Pang, Xulai Zhang

**Affiliations:** 1 Department of Material Dependence, Affiliated Psychological Hospital of Anhui Medical University, Hefei, 230022 China; 2 Anhui Clinical Research Center for Mental Disorders, Anhui Mental Health Center, Hefei, 230022 China; 3 Department of Material Dependence, Hefei Fourth People’s Hospital, Hefei, 230022 China; 4 Department of Epidemiology and Biostatistics, School of Public Health, Anhui Medical University, Hefei, 230032 China; 5 Department of Nursing, Hefei Fourth People’s Hospital, Hefei, 230022 China; 6 Department of Geriatric Psychology, Hefei Fourth People’s Hospital, Affiliated Psychological Hospital of Anhui Medical University, No. 316 Huangshan Road, Hefei, 230022 China; 7 Department of Geriatric Psychology, Anhui Mental Health Center, Hefei, 230022 China

**Keywords:** Alcohol dependence, Vitamin D, Thyroid function, Relationship, Thyroid hormone

## Abstract

**Background:**

Alcohol dependence (AD) results in several medical problems including vitamin D deficiency and thyroid dysfunction. However, the relationship between these two complications remains unclear. The aim of the present study was to explore the relationship between serum vitamin D and thyroid hormone profiles in male patients with AD.

**Methods:**

A total of 117 male patients with AD were enrolled. Vitamin D deficiency was defined as serum concentrations of the main circulating vitamin D, 25-hydroxy vitamin D [25(OH)D], below 50 nmol/L. The AD patients were divided into two groups accordingly: 46 patients with normal vitamin D levels (normal group) and 71 patients with vitamin D deficiency (deficiency group). The levels of thyroid hormone profiles including total triiodothyronine 3 (TT3), total thyroxine 4 (TT4), thyroid stimulating hormone (TSH), free triiodothyronine (fT3), and free thyroxine (fT4) between the two groups were compared. Correlation between the serum levels of 25(OH)D and thyroid hormone profiles was evaluated using simple correlation (Pearson’s correlation) and multivariable analysis using linear regression models.

**Results:**

The prevalence of vitamin D deficiency in male patients with AD is 60.7% (71/117; 95% confidence interval: 51.6–69.1%). Moreover, the serum levels of TT3 (*t* = -2.682, *p* = 0.009), TT4 (*t* = -2.033, *p* = 0.044), fT3 (*t* = -2.986, *p* = 0.003), and fT4 (*t* = -2.558, *p* = 0.012) in deficiency group were significantly higher than those in normal group. Post hoc power analyses showed that the power for fT3 was sufficient (power > 0.80). Furthermore, univariate analysis showed that the serum vitamin D levels were negatively correlated with the TT3 (*r* = -0.189, *p* = 0.044), fT3 (*r* = -0.350, *p* < 0.001), and fT4 (*r* = -0.198, *p* = 0.033) levels, while multivariate analysis indicated that only fT3 was independently related to the serum levels of vitamin D in male patients with AD.

**Conclusions:**

These results suggested that the serum vitamin D levels may be associated with fT3 in male patients with AD.

## Introduction

Alcohol dependence (AD), one of the most frequent and devastating diseases globally, develops following long-term high-level alcohol consumption [1]. It has been well demonstrated that AD results in several medical problems including vitamin D deficiency and thyroid dysfunction [2, 3]. Due to the absence of a singularly efficacious treatment for alcohol abuse, AD and its accompanying medical problems lead to high economic costs to the health care sector and to society [4, 5]. Therefore, it is of great scientific value and clinical significance to deeply explore the internal relationship between the complications of AD.

Vitamin D, a lipid-soluble hormone involved in bone and mineral metabolism, acts by binding with nuclear vitamin D receptor [6]. Clinical data have shown that serum vitamin D concentrations of subjects with alcohol use disorder are 28% lower than those of controls [7]. Vitamin D deficiency is common among patients with alcohol use disorder and is associated with cognitive impairment and osteoporosis [8]. Multiple reasons interact to produce vitamin D deficiency in alcohol-dependent individuals and these include cholestasis or pancreatic insufficiency, poor dietary intake, lack of sunlight exposure, impaired renal synthesis, increased 25-hydroxy vitamin D [25(OH)D] degradation, and direct bowel mucosal lesions [9].

Several studies describe the effects of heavy alcohol consumption on thyroid homeostasis. It has been reported that the toxic effects of heavy and chronic alcohol consumption mainly play a pathological role in thyroid gland dysregulation through employing the gut-brain axis [10]. In animal studies, chronic exposure to ethanol blunted thyrotropic response to cold exposure and resulted in higher levels of thyrotropin-releasing hormone (TRH) mRNA in the paraventricular nucleus (PVN) of the hypothalamus in Sprague-Dawley rats [11]. In addition, recent studies have demonstrated the immunomodulatory properties of vitamin D, thus establishing a correlation between vitamin D deficiency and an increased susceptibility to autoimmune thyroid diseases [12]. However, the causes and potential mechanisms of thyroid dysfunction in patients with AD are still unclear.

Recent evidence has demonstrated a link between serum 25(OH)D levels and thyroid function parameters. A cross-sectional study have showed that hyperthyroidism, hypothyroidism and thyroid peroxidase antibody (TPOAb) positivity were significantly associated with the presence of 25(OH)D deficiency in postmenopausal women with type 2 diabetes mellitus [13]. Moreover, vitamin D supplementation significantly reduced the level of TPOAb in patients with Hashimoto’s thyroiditis [14]. Furthermore, vitamin D deficiency is slightly associated with hypothyroidism in children aged 6–24 months [15]. However, few studies have focused on investigating the correlation between 25(OH)D levels and thyroid dysfunction in male patients with AD.

Considering the potential connection between vitamin D deficiency and thyroid dysfunction, together with the fact that chronic alcohol consumption leads to vitamin D deficiency and thyroid dysfunction, the aim of the present study was to investigate the relationship between serum vitamin D and thyroid hormone profiles in male patients with AD.

## Methodology

### Subjects

This study was conducted at Hefei Fourth People’s Hospital, Anhui Mental Health Center, between July 2021 and October 2022. A total of 117 male subjects with AD were enrolled. All patients met the following inclusion criteria: (1) age 18–70; (2) meet the diagnostic criteria for AD mentioned in the International Classification of Diseases 10th Revision (ICD-10); (3) giving written informed consent to participate in the study. The exclusion criteria were as follows: (1) suffer from mental disorders; (2) diagnosed of other substance dependent diseases; (3) suffering from a major neurological or medical illness. In addition, since liver is an important organ for hormone regulation and vitamins production, these patients were excluded from liver disease other than fatty liver disease. The presence of these disorders was excluded by means of a structured clinical interview. Vitamin D deficiency was defined as serum concentrations of the main circulating vitamin D, 25-hydroxy vitamin D [25(OH)D], below 50 nmol/L. Accordingly, the AD patients were divided into two groups: 46 patients with normal vitamin D levels (normal group) and 71 patients with vitamin D deficiency (deficiency group). This procedure was approved by the ethics committee of Hefei Fourth People’s Hospital (approval number: IRB-HFSY-YJ-LW-LY(2,021,003)) and was conducted according to the principles of the Declaration of Helsinki.

### Collection of clinical data

Demographics, information regarding alcohol drinking, and routine laboratory parameters were collected using a structured questionnaire during face-to-face interviews. The questionnaire contained questions about age, height, body weight, hypertension, years of drinking, years of unordered drinking, amount of alcohol consumed daily, and routine laboratory parameters including total triiodothyronine 3 (TT3), total thyroxine 4 (TT4), thyroid stimulating hormone (TSH), free triiodothyronine (fT3), and free thyroxine (fT4), which were measured using a Siemens Atellica system (Atellica Solution IM1600, SIEMENS Healthineers, Erlangen, Germany).

### Collection of blood sample and measurement of serum 25(OH)D levels

The blood sample was taken from the participants’ vein between 8:00 A.M. and 9:00 A.M. All subjects were fasting while giving blood. Tubes any anticoagulant were used for collecting the samples. The blood samples were immediately centrifuged at 1200 g for 10 min at 4 °C. Extract supernatant as serum sample was collected. The supernatant serum samples were stored immediately at -80 °C until detection. The serum concentrations of 25(OH)D were detected by Adicon Medical Laboratory Center (Adicon clinical laboratories, LTD, Hefei, China) through a chemiluminescence immunoassay analyzer (Mindray CL-2000i, Shenzhen, China).

### Statistical analysis

SPSS (version 17.0; IBM Corp., Armonk, NY, USA) was applied for statistical analysis. Data are presented as mean ± standard error of the mean (SEM), and the level of statistical significance was set at *p*-value < 0.05. Normal distribution for continuous variables was compared by Kolmogorov-Smirnov normality test. Normally distributed continuous data between the normal group and deficiency group were analyzed by Student’s *t*-test, and non-normally distributed data were compared using the Mann-Whitney U-test. Pearson correlation analysis was used to analyze the correlation between the serum levels of vitamin D and thyroid hormone profiles. Multivariate linear regression analysis was performed to evaluate the independent relationship between the serum levels of vitamin D and thyroid hormone profiles, and the models were adjusted for potential confounding factors with stepwise strategies. G*Power (version 3.1.9.2, University of Kiel, Kiel, Germany) was used to calculate the post hoc power of a two-sample *t*-test between the normal group and deficiency group.

## Results

### Demographic values, alcohol-related data, and serum 25(OH)D levels of the normal group and deficiency group

The prevalence of vitamin D deficiency in male patients with AD is 60.7% (71/117; 95% confidence interval: 51.6–69.1%). As shown in Table 1, there were no significant differences in age, BMI, incidence of hypertension, years of drinking, years of unordered drinking, or daily intake between the two groups (all *p* > 0.05). The serum 25(OH)D levels of AD patients with normal vitamin D levels were significantly higher than those of AD patients with vitamin D deficiency (*t* = -10.449, *p* < 0.05).

**Table 1:** Comparison of demographic values, alcohol-related data, and serum 25(OH)D levels between the normal group and deficiency group

Variables	Normal	Deficiency	*t*/*χ*^*2*^	*p*
Age	45.39 ± 1.52	42.45 ± 1.15	1.566	0.120
BMI	22.97 ± 0.40	23.24 ± 0.44	-0.423	0.673
Years of drinking	21.39 ± 1.43	19.99 ± 1.17	0.758	0.450
Years of unordered drinking	3.96 ± 0.47	4.48 ± 0.43	-0.797	0.427
Daily intake (standard drink)	23.81 ± 1.58	24.45 ± 1.26	-0.318	0.751
25(OH)D (nmol/L)	74.81 ± 3.82	32.94 ± 1.20	10.449	< 0.001

### Thyroid hormone profiles of the normal group and deficiency group

As shown in Table 2, the serum levels of TT3 (*t* = -2.682, *p* = 0.009), TT4 (*t* = -2.033, *p* = 0.044), fT3 (*t* = -2.986, *p* = 0.003), and fT4 (*t* = -2.558, *p* = 0.012) in deficiency group were significantly higher than those in normal group. There were no significant differences in the serum TSH levels between the two groups (*t* = -0.627, *p* = 0.532). Post hoc power analyses showed that the power for fT3 was sufficient (power > 0.80) (Table 2).

**Table 2:** Comparison of thyroid hormone profiles between the normal group and deficiency group

Variables	Normal	Deficiency	*t*	*p*	Effect size *d*	Power
TT3 (ng/ml)	1.20 ± 0.05	1.53 ± 0.11	-2.682	0.009	0.460	0.673
TT4 (µg/dl)	17.82 ± 4.91	32.51 ± 5.30	-2.033	0.044	0.374	0.500
TSH (µIU/ml)	2.19 ± 0.24	2.38 ± 0.19	-0.627	0.532	0.116	0.093
fT3 (pg/ml)	3.37 ± 0.14	4.02 ± 0.16	-2.986	0.003	0.553	0.826
fT4 (ng/dl)	2.87 ± 0.66	5.61 ± 0.84	-2.558	0.012	0.464	0.681

### Relationship Between the serum levels of vitamin D and thyroid hormone profiles in male patients with AD

**Fig. 1:**
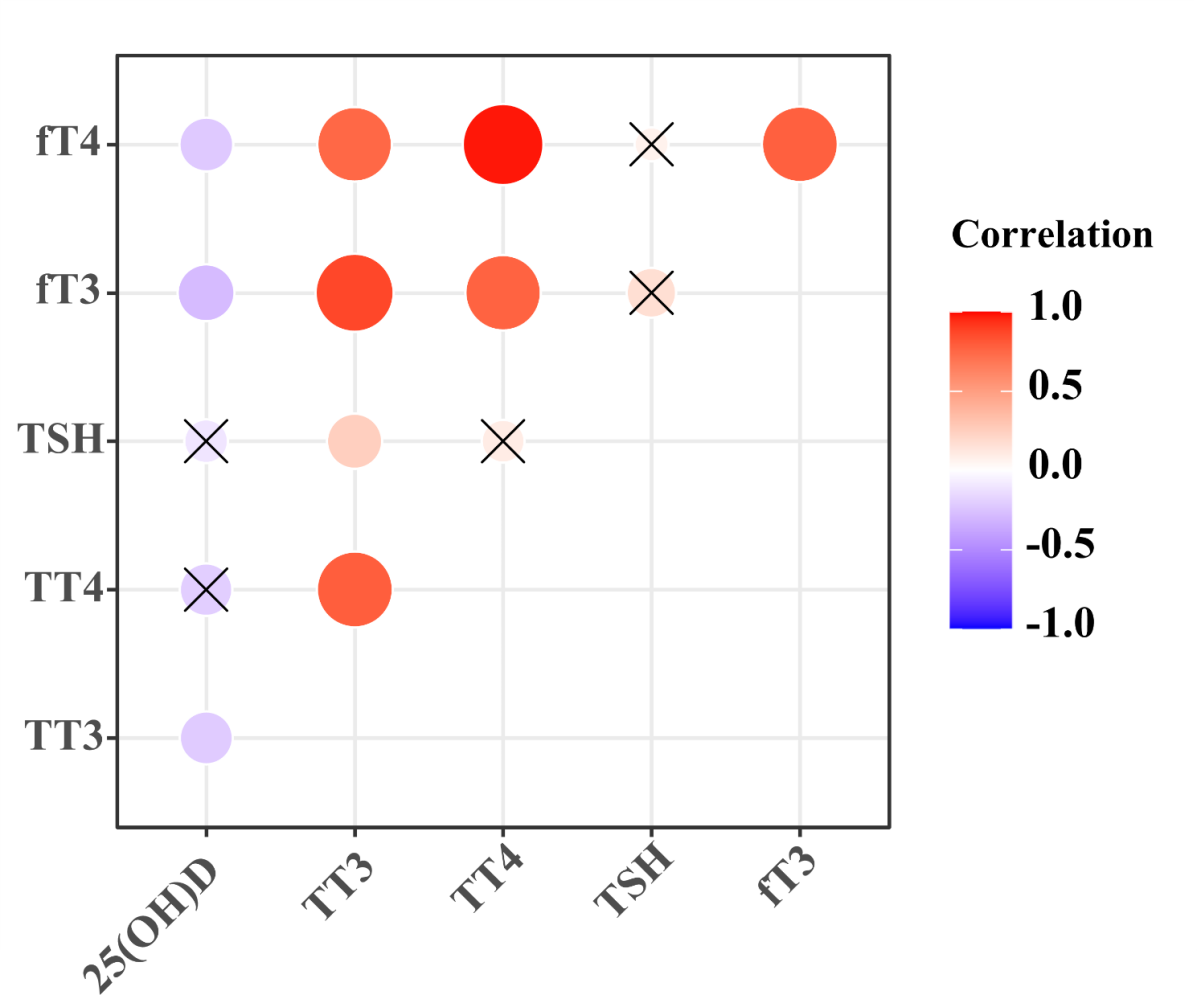
Correlation between the serum 25(OH)D levels and thyroid hormone profiles in male patients with AD. *p < 0.05, **p < 0.01; ×: no significance

Pearson correlation coefficients (*r*) are used to examine the direction, strength, and significance of linear relationships between variables. Univariate analysis showed that there was a negative correlation between serum vitamin D levels and TT3, fT3, or fT4 levels (Fig. 1). The correlation between serum vitamin D levels and TT3 (*r* = -0.189, *p* = 0.044), or fT4 (*r* = -0.198, *p* = 0.033) levels was considerably lower than the correlation between serum vitamin D levels and fT3 (*r* = -0.319, *p* = 0.001). In the multiple linear regression analysis, parameters with independent association with the serum levels of vitamin D (TT3, fT3, and fT4) were included and underwent stepwise adjustment. As shown in the Table 3, from model 1 to model 3, it showed that fT3 levels were negatively associated with the serum levels of vitamin D, which was independent of TT3 and fT4.

**Table 3:** Multivariate linear regression analysis to evaluate the independent relationship between the serum levels of vitamin D and thyroid hormone profiles

Variables	adjusted R-square	*B*	*95% CI*	*p*
Model 1	0.076			
fT3		-6.682	-10.900, -2.465	0.002
Model 2	0.071			
fT3		-9.103	-17.657, -0.549	0.037
T3		11.022	-14.738, 28.965	0.520
Model 3	0.062			
fT3		-8.725	-17.992, 0.542	0.048
T3		7.732	-14.944, 30.408	0.500
fT4		-0.139	-1.410, 1.133	0.829

Model 1: fT3, adjusted for T3 and fT4

Model 2: fT3 + T3, adjusted for fT4

Model 3: fT3 + T3 + fT4

## Discussion

The present study firstly investigated the relationship between serum vitamin D and thyroid hormone profiles in male patients with AD. Three main findings emerged from the present study. First, the prevalence of vitamin D deficiency in male patients with AD is 60.7% (71/117). Second, the levels of TT3, TT4, fT3, and fT4 in AD patients with vitamin D deficiency were significantly higher than those of AD patients with normal vitamin D levels. Third, the serum vitamin D levels were negatively correlated with the TT3, fT3, and fT4 levels in male patients with AD.

Several lines of evidence have demonstrated that vitamin D deficiency is more present in alcohol-dependent individuals. It has been suggested that vitamin D deficiency in alcohol-dependent individuals is a result of reduced light exposure, poor nutrition, alcohol-induced liver damage, and malabsorption [16]. A cross-sectional study found that the prevalence of vitamin D deficiency in 174 patients with AD was 64% [17]. Similarly, in the present study, the prevalence of vitamin D deficiency in male patients with AD was 60.7%, further confirming the high incidence of vitamin D deficiency in patients with AD. In addition, another study have reported that serum 25(OH)D concentrations in the AD adolescents were significantly lower than in non-AUD adolescents [18]. Nearly half of AD adolescents (48.8%) were classified as being vitamin D deficient [18]. Given the escalating prevalence of heavy adolescent drinking as a pressing public health concern, coupled with the crucial role of sufficient vitamin D in facilitating optimal skeletal development during adolescence, it is imperative to implement efficacious intervention strategies for individuals diagnosed with AD, with particular emphasis on AD adolescents.

There is a link between vitamin D levels and thyroid hormone profiles. More recently, a cross-sectional observational study was conducted to determine whether there is a link between vitamin D levels and thyroid hormone sensitivity in 8,126 participators from the National Health and Nutrition Examination Survey (NHANES) database [19]. It has been found that Thyroid Feedback Quantile-based Index (TFQI) and fT3/fT4 were nonlinearly correlated with vitamin D levels, meaning that both the central and peripheral thyroid hormone sensitivity were nonlinearly correlated with vitamin D [19]. Another cross-sectional study was performed to explore the association of vitamin D with thyroid hormone sensitivity in an euthyroid adult Chinese population (3143 subjects) [20]. The results showed that serum fT4, fT4, and TSH were higher in subjects with vitamin D deficiency than those with sufficient vitamin D, and serum 25(OH)D levels were significantly negatively correlated with fT3, fT4, and TSH levels in euthyroid adults [20]. The present study firstly compared the thyroid hormone profiles between AD patients with normal vitamin D levels and with vitamin D deficiency. The results showed that in the deficiency group, serum TT3, TT4, fT3, and fT4 levels were significantly higher than in the normal group. Further analysis was conducted on the correlation between vitamin D levels and thyroid hormone profiles, and the results showed that serum vitamin D levels were negatively correlated with TT3, fT3, and fT4, respectively, and fT3 was found to be independently related to vitamin D levels. These findings demonstrated a close correlation between thyroid hormone profiles, particularly fT3, and vitamin D insufficiency in male AD patients.

The underlying mechanisms that may explain these associations have not been well understood. It has been indicated that only vitamin D-deficient BALB/c mice (BALB/c is an albino, laboratory-bred strain of the house mouse and is among the most widely used inbred strains used in animal experimentation) may develop persistent hyperthyroidism after being immunized with the TSH receptor [21]. Another animal study has found that an increase in T3, T4, and TSH may result in an increase in vitamin D concentration in rats [22]. More related experiments should be conducted to further elucidate the mechanism of vitamin D deficiency and thyroid hormone interactions in male patients with AD.

Several limitations should be highlighted. First, the small sample size and single-center design of the current study may indicate sampling bias. Second, the causal relationship between vitamin D deficiency and thyroid function in male patients with AD cannot be examined in this cross-sectional study. Third, psychological assessments such as offensive attitude, cognitive dysfunctions, psychological craving, anxiety and depression symptoms and their correlation with the serum levels of vitamin D and thyroid hormone profiles in male patients with AD were not evaluated in this study. Fourth, since the vast majority of Chinese patients with AD are male patients, our hospital has only set up male inpatient areas. Thus, only male patients with AD were included in the present study. The inability to examine gender differences may be considered a limitation.

## Conclusion

The present study suggests that serum vitamin D levels may be associated with fT3 dysfunction in male AD patients. A multicentric study is clearly required to validate this potential relationship, and longitudinal studies are needed to investigate the causal relationship between vitamin D deficiency and thyroid hormone profiles in male patients with AD .

## Data Availability

The dataset used and analyzed during the current study are available from the corresponding author on reasonable request.
